# Comparing Adherence with Best Practices in End-of-Life Care After Implementing the End-of-Life Order Set: A Quality Improvement Project in an Ottawa Academic Hospital

**DOI:** 10.1089/pmr.2022.0070

**Published:** 2023-04-14

**Authors:** Grace Warmels, Anne Roberts, John Haddad, Marie-Hélène Chomienne, Shirley H. Bush, Valerie Gratton

**Affiliations:** ^1^ Division of Palliative Care, Department of Medicine, University of Ottawa, Ottawa, Ontario, Canada.; ^2^ Clinical Epidemiology Program, Ottawa Hospital Research Institute, Ottawa, Ontario, Canada.; ^3^ Department of Palliative Care, Montfort Hospital, Ottawa, Ontario, Canada.; ^4^ Department of Family Medicine, University of Calgary, Calgary, Alberta, Canada.; ^5^ Faculty of Medicine, University of Ottawa, Ottawa, Ontario, Canada.; ^6^ Institut du Savoir Montfort, Ottawa, Ontario, Canada.; ^7^ Bruyère Research Institute, Ottawa, Ontario, Canada.; ^8^ Department of Family Medicine, Montfort Hospital, Ottawa, Ontario, Canada.

**Keywords:** comfort measures, end of life, order set, palliative care

## Abstract

**Background:**

Physicians in acute care require tools to assist them in transitioning patients from a “life prolonging” approach to “end-of-life care,” and standardized order sets can be a useful strategy. The end-of-life order set (EOLOS) was developed and implemented in the medical wards of a community academic hospital.

**Objective:**

To compare adherence with best practices in end-of-life care after implementing the EOLOS.

**Methods:**

We conducted a retrospective chart review of admitted patients with expected deaths in the year preceding EOLOS implementation (“before EOLOS” group), and in the 12 to 24 months following EOLOS implementation (“after EOLOS” group).

**Results:**

A total of 295 charts were included: 139 (47%) in the “before EOLOS” group and 156 (53%) in the “after EOLOS” group, of which 117/156 charts (75%) had a completed EOLOS. The “after EOLOS” group demonstrated more “do not resuscitate” orders and more written communication to team members about comfort goals of care. There was a decrease in nonbeneficial interventions in the last 24 hours of life in the “after EOLOS” group: high-flow oxygen, intravenous antibiotics, and deep vein thrombosis/venous thromboembolism prophylaxis. The “after EOLOS” group demonstrated increased prescription of all common end-of-life medications, except for opioids, which had a high preexisting rate of prescription. Patients in the “after EOLOS” group showed a higher rate of spiritual care and palliative care consult team consultation.

**Conclusion:**

Findings support standardized order sets as a good framework allowing generalist hospital staff to improve adherence to established palliative care principles and improve end-of-life care of hospital inpatients.

## Introduction

How and where we die matters to patients.^1–5^ We know that a high percentage of people die in hospitals. An international study looking at place of death in 45 populations, confirmed that 54% of deaths occurred in hospitals.^6^ In Canada, this number is closer to 59%.^7^

Generalist physicians, such as family doctors and internal medicine specialists,^8,9^ provide the majority of inpatient end-of-life care. Within this group, there are varying levels of expertise^10,11^ and managing end-of-life in an acute care setting brings additional challenges when compared with dying at home or in a hospice setting. The well-known SUPPORT trial^12^ published 30 years ago highlighted shortcomings in communication, high frequency of aggressive treatments, and suboptimal symptom management in hospitalized patients at the end of their life.

In recent years, numerous studies^13–17^ reconfirm that in an acute care setting, intensive treatments are often ongoing for patients in their last hours of life. These interventions include parental hydration, intravenous (IV) antibiotics, transfusions, surgery, and life support treatments (e.g., mechanical ventilation), which are nonbeneficial and contribute to discomfort.^17^ This is contrary to what patients have defined as a quality death, which involves adequate symptom management and avoidance of prolonging dying phase.^18,19^ In a literature review,^19^ defining a “good death” encompasses respect of preferences for the dying process such as control over the circumstances of the dying and preparation for death (reported in 94% of articles); relieving suffering (81%); and supporting emotional well-being (64%).

Furthermore, studies have demonstrated poor documentation of patients' end-of-life wishes.^12,20,21^ Incomplete documentation of goals is linked to inconsistent messaging among team members and to suboptimal care delivery. Assuring that teams focus on patient-specific goals and care plans promotes continuity and satisfaction.^18^

To address these gaps in providing quality care for patients dying in acute care hospitals, strategies to support the transition from “disease modifying treatment” to “comfort care” are needed. Standardized order sets are clinical decision support tools that aim to help physicians prescribe appropriate treatments using a predefined set of recommendations linked to evidence-based practice.^22^ They have become common in clinical practice including in palliative care and have proven to increase physician comfort while prescribing less familiar drugs, to improve symptom management by reducing errors^23–25^ and variability of orders among clinicians.^26^

In our hospital in Ottawa, Ontario, the palliative care consult team (PCCT) noted anecdotally that practices around managing end-of-life varied greatly, often dependent on where physicians had trained.^27^ The PCCT was approached by physicians, nursing leaders, and the medical chief of staff requesting the development of a standardized order set as a resource for providing end-of-life care. In response, the PCCT reviewed the evidence^21,24–26,28^ for using standardized order sets, aware of concerns that order sets have been compared with “tick box” exercises.^26^ Satisfied, we aimed to develop an order set that integrated best practices for end-of-life care.

## Development of the End-of-Life Order Set

Standardized order sets were already common within our hospital culture for conditions such as pneumonia, stroke, and heart failure. In September 2015, the PCCT and a pharmacist developed the end-of-life order set (EOLOS) (Supplementary Appendix SA1). The team was guided by examples of “comfort measures order sets (CMOS)” obtained via a literature review, looking specifically for articles that included their copy of the order set.^24,26,28–30^ The revision process included regular interdisciplinary group meetings, feedback from our key institutional committees, and consultation with local experts. The EOLOS was also beta-tested by the PCCT before its formal implementation.

The EOLOS is a bilingual (French and English), two-sided, paper-based document designed to be completed by a physician. The first page of the EOLOS prompts the initiation of nonpharmacological interventions (e.g., the insertion of a urinary catheter as needed) and cessation of nonbeneficial medical interventions (e.g., IV fluids). There is an option to request a palliative or spiritual care consultation. Physicians then prescribe standard palliative care medications. Certain medications, such as opioids and antipsychotics (haloperidol, methotrimeprazine), with a need for more flexibility around dose and frequency, are completed by the physician. We opted to prefill the dose and frequency for other medications with less variability in their start doses (anticholinergics, midazolam, and lorazepam for seizures).

The second page features “clinical pearls” as an educational resource, including suggested start doses for opioids, haloperidol, and methotrimeprazine; indications or rationale for medications (e.g., haloperidol is a potent antiemetic); and tips on various end-of-life interventions such as hydration, oxygen use, and managing secretions.

Three criteria are required for a physician to initiate the EOLOS: (1) a “do not resuscitate” (DNR) order; (2) the patient is dying of a life-limiting illness, prognosis hours/days (Palliative Performance Scale ≤30%^31–33^); and (3) the care goals have been discussed with the patient or the substitute decision maker.

## Aim

To compare adherence with best practices in end-of-life care after implementing the EOLOS in our hospital.

## Methods

### Setting and context

The setting is a 289-bed community academic hospital in Ottawa, Canada, with ∼140 medicine beds. Hospitalists are responsible for most inpatients at end of life, and the PCCT, comprising a palliative care nurse and physician, is available on weekdays. The hospital transitioned from a paper-based medical record system to an electronic medical system in July 2015, just before the implementation of the EOLOS.

### Ethics approval

Ethics approval was received from the Montfort Research Ethics Board (#18-19-08-021; October 26, 2018).

### Implementation of the EOLOS

The EOLOS was implemented in September 2015, as part of a multiprong approach to improve end-of-life care at our institution. Along with the EOLOS, we designed education sessions for physicians and nursing staff, as well as an end-of-life booklet for families. The staff physicians, medical learners (medical students and residents), and nurses received separate training sessions from the PCCT, detailed in Table 1. A goal of the educational plan was to integrate ongoing clinical and academic activities, rather than add to the busy workload of clinicians and learners. In addition to formal teaching sessions, informal mentoring (coaching at the bedside; reviewing EOLOS prescription with clinicians 1:1) from the PCCT was delivered to nurses and physicians to assure proper understanding and use of the EOLOS.

**Table 1.** Project Time Line

Date	Milestone
2014–2015	Development of the EOLOS
September 2015	EOLOS finalized
September 2015–September 2016	Education and rollout of the EOLOS to MDs, medical learners (students and residents), and nursing staff: Staff MDs: 30 minutes presentation by PC physician (VG) during departmental meeting to review the EOLOS, including criteria for initiation, a brief review of the pharmacology for common end-of-life symptoms, and clinical pearls Family medicine residents: 90-minute lecture by a PC physician (VG) on the EOLOS and end-of-life symptom management Medical students (third year): 90-minute lecture by a PC physician (VG) on the EOLOS and end-of-life symptom management (repeated q2 months to deliver to all students—total 6 lectures) Nurses: during the first week of rollout, daily presentation by PC nurse specialist at nursing rounds to introduce the EOLOS
May 2016–February 2017	Formal teaching session to nurses: 18 teaching sessions given by PC nurse specialist, with ∼275 total attendees
January 2019	Development of data extraction template
January–July 2019	Chart review data collection for both before/after EOLOS groups
October–November 2019	Data analysis

EOLOS, end-of-life order set; MD, medical doctor; PC, palliative care; VG, Valerie Gratton.

### Evaluation method

This quality improvement project used a retrospective chart review before and after the implementation of the EOLOS.

### Retrospective chart review

We conducted a retrospective chart review of admitted medicine patients with expected deaths that occurred between September 1, 2014, and September 1, 2015 (i.e., 12 months before EOLOS implementation—the “before EOLOS” group), and between September 1, 2016, and September 1, 2017 (i.e., 12–24 months following the EOLOS's implementation—the “after EOLOS” group).

The charts of all deceased patients from the medical wards during the “before EOLOS” and “after EOLOS” periods were reviewed. An experienced palliative care nurse reviewed the charts and determined eligibility based on the inclusion and exclusion criteria. A death was determined to be “expected” if indicated in the progress notes or orders (e.g., “comfort care,” “palliative patient,” or “expected death”); a completed DNR order on its own was insufficient. Patients were excluded if they died outside the medical wards. Unexpected deaths on medical wards, deaths occurring when the patient's goals were for active medical management, or charts with privacy restrictions were also excluded. Patients who had received a PCCT consult were not excluded, as this was part of “usual care.”

The EOLOS was considered completed if it was present and signed by a physician in the patient's chart. Not all sections were required to be “checked off” for them to be deemed completed, leaving room for individual patient care needs and physician practice.

#### Data collection and evaluation

A data extraction spreadsheet template was created by a palliative care nurse and physician. After piloting the template on the first 20 charts, iterative changes were made. Data were extracted from both paper charts (“before EOLOS” group) and patients' electronic medical records (“after EOLOS” group).

This palliative care nurse and a medical student (trained by the palliative care nurse) extracted the following data: (1) sociodemographic data, including age, sex, date of death, and location before hospital admission; (2) primary diagnosis; and (3) clinical data for 48 hours before death, pertaining to accepted palliative care principles for patients at the end of life:

1. Documenting and communicating care goals: DNR orders and physician documentation communicating a palliative approach and expected death to allied health (accepted examples: documentation in the medical orders of “palliative/comfort care,” “expected death,” the presence of a signed EOLOS). To qualify, it was imperative that communication to the entire care team be clear; a mention of an expected death in the doctor's progress notes, for example, would not suffice.
2. Nonbeneficial interventions including high-flow oxygen (not including oxygen by nasal prongs), blood transfusions, blood glucose monitoring, blood tests, diagnostic imaging, IV fluids, IV antibiotics, injectable deep vein thrombosis prophylaxis, and Rapid Assessment of Critical Events calls.
3. Medication availability: written orders (both “scheduled” and “as needed”) for subcutaneous opioids, benzodiazepines, antipsychotics, anticholinergics, and rectal acetaminophen.

### Analysis

Descriptive statistics were calculated for all variables. Continuous variables were presented as means and standard deviations. Categorical variables were presented as total numbers and percentages. Sociodemographic and characteristics were compared for differences between the before and after EOLOS implementation groups. Statistical analysis included unpaired t-test for continuous variables and chi-square for categorical variables to determine the significance of means and the significance of group characteristics. A *post hoc* pairwise z-test with Bonferroni correction was performed for significant chi-square results, with more than two categories, to determine which percentages differed significantly from each other. Data were analyzed using IBM SPSS Statistics for Windows, Version 22.^34^ A *p*-value of 0.05 indicated statistical significance.

## Results

A total of 295 charts met the inclusion criteria, with 139 (47%) in the “before EOLOS” group and 156 (53%) in the “after EOLOS” group. Numbers of charts were slightly lower when looking at nonbeneficial medical treatments 24 to 48 hours before death (132 “before EOLOS” and 130 “after EOLOS”), reflecting the fact that some patients arrived at hospital with death imminent, and died within 24 hours of admission. There was no significant difference between patients' age, sex, or location before hospital admission. Primary diagnosis (e.g., cancer; respiratory [e.g., COPD]; cardiac [e.g., congestive heart failure]) between the groups was similar, apart from an increased number of “other” conditions and a decreased hematological diagnosis in the “after EOLOS” group (Table 2).

**Table 2.** Sociodemographic Data of Included Patient Charts

	“Before” EOLOS implementation ***n*** = 139 (%)	“After” EOLOS implementation ***n*** = 156 (%)	χ^2^	***p***
Age (years ± standard deviation)	82.7 ± 10.9	82.9 ± 10.5		0.879
Median age (years)	84	85		
Range (years)	38–101	52–103		
Location before hospital admission			2.813	0. 421
Home	65 (47)	73 (47)		
Retirement home	34 (24)	49 (31)		
Long-term care	37 (27)	31 (20)		
Other	2 (1)	3 (2)		
Unknown	1 (1)	0 (0)		
Sex			0.021	0.885
Male	68 (49)	75 (48)		
Female	71 (51)	81 (52)		
Primary diagnosis (up to 2 per patient)			15.032	0.027
Cancer	27^a^ (19)	27^a^ (17)		
Respiratory	20^a^ (14)	13^a^ (8)		
Cardiac	21^a^ (15)	18^a^ (12)		
Neurologic	24^a^ (17)	30^a^ (19)		
Hepatic	2^a^ (2)	1^a^ (0.5)		
Dementia	24^a^ (17)	36^a^ (23)		
Hematology	**7^a^ (5)**	**1^b^ (0.5)**		
Other	**15^a^ (11)**	**32^b^ (20)**		

Each superscripted letter indicates a subset of group categories whose column proportions do not differ significantly from each other at the 0.05 level. Within each row, percentages that do not share a superscript are significantly different (indicated in bold). Other: Mostly refers to patients with extensive comorbidities where no primary diagnosis could be clearly established.

In the “after EOLOS” group, 117/156 charts (75%) had a completed EOLOS. Our team opted to analyze the data of the “after EOLOS” group jointly (patients with and without EOLOS), as this represents the systemic impact of EOLOS as a whole.

In comparing the two groups, we noted that documentation of DNR increased (from 90% to 100%, *p* ≥ 0.001). The evidence of clear documentation communicating comfort care and expected death increased from 72% to 92%, *p* ≥ 0.001.

There was a statistically significant decrease in the following nonbeneficial interventions in the last 24 hours of life in the “after EOLOS” group: high-flow oxygen (decreased from 18% to 10% of patients, *p* = 0.036), IV antibiotics (decreased from 23% to 11%, *p* = 0.005), and deep vein thrombosis prophylaxis (decreased from 19% to 10%, *p* = 0.038). Considering the last 48 hours of life, the only significant reduction between the “before EOLOS” and “after EOLOS” groups was seen in the use of IV fluids (from 24% to 13%, *p* = 0.018).

The “after EOLOS” group demonstrated improved medication prescriptions, including antipsychotics (69% vs. 84%, *p* = 0.002), benzodiazepines (63% vs. 86%, *p* ≥ 0.001), anticholinergics (70% vs. 87%, *p* ≥ 0.001), and acetaminophen (60% vs. 87%, *p* ≥ 0.001). Opioid prescription rates were slightly increased in the “after EOLOS” group, but not significantly.

Patients in the “after EOLOS” group showed a higher rate of consultation requests for spiritual care (from 41% to 62% *p* < 0.001) and to the PCCT (from 57% to 74%, *p* = 0.002; Table 3).

**Table 3.** Documentation of Clinical Care and Communication

	Before EOLOS implementation ***n*** = 139 (%)	After EOLOS implementation ***n*** = 156 (%)	***p***
DNR present	125 (90)	156 (100)	**<0.001**
Palliative care consult	79 (57)	116 (74)	**0.002**
Spiritual care consult	57 (41)	96 (62)	**<0.001**
Physician documentation clearly communicating to allied health palliative care goals and anticipated death	100 (72)	143 (92)	**<0.001**
Presence of nonbeneficial medical treatments (0–24 hours before death)			
High-flow oxygen	25 (18)	15 (10)	**0.036**
Blood transfusion	1 (0.7)	1 (0.6)	—
Blood glucose monitoring	16 (12)	8 (5)	**0.045**
Bloodwork (any)	24 (17)	18 (12)	0.16
Imaging	18 (13)	15 (10)	0.364
IV fluids	1 (0.7)	0 (0)	**0.001**
IV antibiotics	32 (23)	17 (11)	**0.005**
Venous thromboembolism prophylaxis	26 (19)	16 (10)	**0.038**
RACE call	4 (3)	3 (2)	0.710

	**Before EOLOS implementation** ***n* = 132**	**After EOLOS implementation** ***n* = 150**	
Presence of nonbeneficial medical treatments (24–48 hours before Death)			
High-flow oxygen	22 (17)	20 (13)	0.433
Blood transfusion	1 (0.8)	0 (0)	0.468
Blood glucose monitoring	21 (16)	20 (13)	0.54
Bloodwork (any)	46 (35)	48 (32)	0.63
Imaging	28 (21)	28 (19)	0.593
IV fluids	32 (24)	20 (13)	**0.018**
IV antibiotics	36 (27)	37 (25)	0.618
Venous thromboembolism Prophylaxis	32 (24)	26 (17)	0.152
RACE call	7 (5)	7 (5)	0.806
Medications prescribed in the last 48 hours of life (including both “scheduled” and “as needed”)			
Opioid	133 (96)	154 (99)	0.154
Antipsychotic	96 (69)	131 (84)	**0.002**
Benzodiazepine	87 (63)	143 (86)	**<0.001**
Anticholinergic	97 (70)	136 (87)	**<0.001**
Acetaminophen	84 (60)	135 (87)	**<0.001**

Bold = statistically significant p value ≤ 0.05.

DNR, do not resuscitate; IV, intravenous; RACE, Rapid Assessment of Critical Events.

## Discussion

Our project aimed to compare adherence with best practices in end-of-life care,^28^ after implementing the EOLOS in an academic hospital; in particular, communication of a “comfort” approach to team members, discontinuation of nonbeneficial medical interventions, and prescription of standard symptom management medications. These principles were chosen to reflect priorities outlined in the literature regarding patients' preferences at their end of life.^18,19,35^

Consistent with previous authors,^21,23,28^ we found an overall improvement in the documentation of care goals, discontinuation of nonbeneficial medical interventions, and the prescribing of common symptom management medications in the last days of life. First, the outcome of clearer communication regarding care goals should result in the delivery of more consistent messaging to family, care that is more aligned with the patient's wishes, improved continuity, and overall satisfaction.^36^ Acknowledgment that the patient is at end of life might allow timely exploration of the patient and family's preferences surrounding the dying phase, preparation for the death, and improved emotional support—aspects highlighted as important for a “good death.”^19^

Second, we found that the rate of nonbeneficial medical interventions decreased after initiating the EOLOS, mainly in the last 24 hours of a patient's life. This may indicate that earlier signs of imminent death (e.g., in the last 48–72 hours of life) remain poorly recognized within acute care hospitals. A recent study^37^ concluded that the majority of providers caring for hospitalized patients at the end of life felt that comfort care was initiated too late, resulting in moral distress for health care providers. It is important to acknowledge that a limitation of the EOLOS is that imminent death must be recognized for it to be useful.

Third, we demonstrated an improved prescription of standard end-of-life medications after implementing the EOLOS; this may have led to improved symptom management. However, actual medication administration or its effects were not part of the scope of this study. The increased rate of palliative medication prescriptions was significant in every category, except for opioids, which was not significant due to a high preexisting rate of opioid prescription at the end of life. This differs from prior studies that demonstrated improvements in opioid prescription with the use of CMOS.^28^ Regarding antipsychotics, our findings are consistent with a recent study evaluating the pharmacological management of terminal delirium in dying patients, demonstrating that patients with a CMOS in place were more comfortable than those without.^38^ Another study^26^ demonstrated a decreased need for medication adjustment for end-of-life symptoms when a CMOS was used. Therefore, CMOS, along with other institutional strategies, likely contribute to more appropriate pharmacological management at the end of life.^23–26,28,29,38^

Notably, one year after the EOLOS's introduction, 75% of patients with an expected mortality rate were prescribed the EOLOS, which indicates excellent uptake at our institution. We opted to analyze the data of the “after EOLOS” group jointly, without separating the patients without completed EOLOS, as this represents the systemic impact of the EOLOS on our institution. For example, even if a patient was not directly prescribed the EOLOS, they might have benefited from the teaching the provider had received.

As other studies have found,^26,28,39,40^ standardized order sets can be useful tools in promoting systemic change within hospitals. The 75% uptake in our hospital contrasts with previous studies where 20% to 67% of those referred to palliative or end-of-life care had standardized orders initiated.^26,29,30^ This success might be explained partly by the culture of our hospital, where order sets are common practice, and by the fact that the providers themselves identified the need for the EOLOS. The initial implementation strategy and education program, as well as ongoing education and mentoring from the PCCT, certainly contributed. In fact, we noted that the EOLOS was quickly embraced by the nursing staff, who strongly advocated its use.

From this, we extrapolate that the EOLOS gave the nurses clear messaging regarding care goals, allowing them to take charge of the end-of-life care plan while giving them the tools to manage the expected symptoms. This points to the EOLOS's significant impact on end-of-life care in our hospital.

### Sustainability

Education sessions are ongoing to ensure that all cohorts of trainees receive proper EOLOS training, and new medical staff are provided with informal, individual teaching regarding the EOLOS by the PCCT. All family medicine residents assigned to our institution receive a 1.5-hour lecture by a palliative care physician on the EOLOS. Newly hired nurses are taught separately by the palliative care nurse during their orientation training (60-minute session; approximately eight times per year).

With our institution's transition to electronic prescriptions, the EOLOS was easily adapted to fit our electronic medical records.

### Project strengths

The strengths of this project include its considerable sample size, multiple endpoints, and longitudinal sampling (performed over one year for each group). Another strength was the multimodal intervention, which included the EOLOS and ongoing education, aimed at both physicians and nurses. Finally, the chart eligibility was determined by one person with expertise in the matter (palliative care nurse), and the data extraction was performed by only two people, assuring consistency.

### Project limitations

This project was a retrospective study in a single center. We did not evaluate whether the prescription of medications for symptom management translated into improved symptom control. Furthermore, we evaluated communication among the care team but not among patients or their families. Our hospital's transition to electronic medical records in July 2015 led to a slightly higher number of digitized medical charts meeting the inclusion criteria in the “after EOLOS” group.

## Conclusion

In conclusion, this quality improvement project supports using standardized order sets in palliative care. Although they are not a substitution for quality, individualized palliative care, standardized order sets represent a framework allowing generalist hospital staff to improve adherence to established palliative care principles. This project was not designed to evaluate patient outcomes but does point to improved patient care at the end of life. Future work should further evaluate the impact of the EOLOS on symptom management and whether it does indeed contribute to a more peaceful death and greater family satisfaction.

## Supplementary Material

Supplemental data

## Supplementary Material

Supplementary Appendix SA1

## Abbreviations Used

CMOS: comfort measures order sets
DNR: do not resuscitate
EOLOS: end-of-life order set
IV: intravenous
MD: medical doctor
PC: palliative care
PCCT: palliative care consult team
RACE: Rapid Assessment of Critical Events

## References

[1] World Health Organization. WHO Palliative Care Fact Sheet. n.d.

[2] Canadian Institute for Health Information. Access to Palliative Care in Canada. Ottawa, ON: CIHI; 2018 n.d.;67.

[3] Higginson I, Sen-gupta G. Place of care in advanced cancer: A qualitative systematic literature review of patient preferences. n.d.; doi: 10.1089/jpm.2000.3.287 15859670

[4] Hoare S, Morris ZS, Kelly MP, et al. Do patients want to die at home? A systematic review of the UK literature, focused on missing preferences for place of death. PLoS One 2015;10(11):e0142723; doi: 10.1371/journal.pone.0142723 26555077 PMC4640665

[5] Gomes B, Higginson IJ, Calanzani N, et al. Preferences for place of death if faced with advanced cancer: a population survey in England, Flanders, Germany, Italy, the Netherlands, Portugal and Spain. Ann Oncol 2012;23(8):2006–2015; doi: 10.1093/annonc/mdr602 22345118

[6] Broad JB, Gott M, Kim H, et al. Where do people die? An international comparison of the percentage of deaths occurring in hospital and residential aged care settings in 45 populations, using published and available statistics. Int J Public Health 2013;58(2):257–267; doi: 10.1007/s00038-012-0394-5 22892713

[7] Government of Canada SC. Deaths, by Place of Death (Hospital or Non-Hospital). 2020. Available from: https://www150.statcan.gc.ca/t1/tbl1/en/tv.action?pid=1310071501 [Last accessed: January 15, 2021].

[8] Doré M. Hospitalists and family physicians. Can Fam Physician 2017;63(6):429–429. PMC5471077 28615389

[9] Canadian Society of Hospitalist Medicine. About CSHM | Canadian Hospitalist. n.d. Available from: https://canadianhospitalist.ca/content/about-cshm/ [Last accessed: January 25, 2022].

[10] Canadian Hospice Palliative Care Association. Discussion Documents | The Way Forward. 2015. Available from: https://www.hpcintegration.ca/resources/discussion-papers/palliative-approach-to-care.aspx [Last accessed: January 15, 2021].

[11] Clark JM, Lurie JD, Claessens MT, et al. Factors associated with palliative care knowledge among internal medicine house staff. J Palliat Care 2003;19(4):253–257. 14959595

[12] SUPPORT. A controlled trial to improve care for seriously ill hospitalized patients. The study to understand prognoses and preferences for outcomes and risks of treatments (SUPPORT). The SUPPORT Principal Investigators. JAMA 1995;274(20):1591–1598. 7474243

[13] Jox RJ, Schaider A, Marckmann G, et al. Medical futility at the end of life: the perspectives of intensive care and palliative care clinicians. J Med Ethics 2012;38(9):540–545; doi: 10.1136/medethics-2011-100479 22562948

[14] Jani B, Blane D, Browne S, et al. Identifying treatment burden as an important concept for end of life care in those with advanced heart failure. Curr Opin Support Pall Care 2013;7(1):3–7; doi: 10.1097/SPC.0b013e32835c071f 23196381

[15] Piers RD, Van den Eynde M, Steeman E, et al. End-of-life care of the geriatric patient and nurses' moral distress. J Am Med Dir Assoc 2012;13(1):80.e7-80.e13; doi: 10.1016/j.jamda.2010.12.014 21450237

[16] Kwok AC, Semel ME, Lipsitz SR, et al. The intensity and variation of surgical care at the end of life: a retrospective cohort study. Lancet 2011;378(9800):1408–1413; doi: 10.1016/S0140-6736(11)61268-3 21982520

[17] Cardona-Morrell M, Kim J, Turner R, et al. Non-beneficial treatments in hospital at the end of life: a systematic review on extent of the problem. Int J Qual Health Care 2016;28(4):456–469; doi: 10.1093/intqhc/mzw060 27353273

[18] Heyland DK. What matters most in end-of-life care: perceptions of seriously ill patients and their family members. CMAJ 2006;174(5):627–633; doi: 10.1503/cmaj.050626 16505458 PMC1389825

[19] Meier EA, Gallegos JV, Thomas LPM, et al. Defining a good death (successful dying): Literature review and a call for research and public dialogue. Am J Geriatr Psychiatry 2016;24(4):261–271; doi: 10.1016/j.jagp.2016.01.135 26976293 PMC4828197

[20] Blinderman CD, Billings JA. Comfort care for patients dying in the hospital. N Engl J Med 2015;373(26):2549–2561; doi: 10.1056/NEJMra1411746 26699170

[21] Bailey FA, Burgio KL, Woodby LL, et al. Improving processes of hospital care during the last hours of life. Arch Intern Med 2005;165(15):1722–1727; doi: 10.1001/archinte.165.15.1722 16087819

[22] Grissinger M. Guidelines for standard order sets. Pharm Ther 2014;39(1):10–50. PMC3956384 24672209

[23] Luhrs CA, Meghani S, Homel P, et al. Pilot of a pathway to improve the care of imminently dying oncology inpatients in a Veterans Affairs Medical Center. J Pain Symptom Manage 2005;29(6):544–551; doi: 10.1016/j.jpainsymman.2005.02.010 15963862

[24] Jarabek BR, Jama AA, Cha SS, et al. Use of a palliative care order set to improve resident comfort with symptom management in palliative care. Palliat Med 2008;22(4):343–349; doi: 10.1177/0269216308090169 18541638

[25] Wiprud R. 30 standardized hospital admission orders. Fam Pract Manag 2001;8(9):49–51. 11685869

[26] Lau C, Stilos K, Nowell A, et al. The comfort measures order set at a tertiary care academic hospital: Is there a comparable difference in end-of-life care between patients dying in acute care when CMOS is utilized? Am J Hosp Palliat Care 2018;35(4):652–663; doi: 10.1177/1049909117734228 28982259

[27] Dufort-Rouleau C, Martin B, Barré V, et al. Conformity in prescription and administration of respiratory distress protocols in a tertiary care hospital in the province of quebec: RELIEVE study. J Palliat Care 2020;35(1):21–28; doi: 10.1177/0825859719835555 30898064

[28] Bailey FA, Williams BR, Woodby LL, et al. Intervention to improve care at life's end in inpatient settings: the BEACON trial. J Gen Intern Med 2014;29(6):836–843; doi: 10.1007/s11606-013-2724-6 24449032 PMC4026508

[29] Walker KA, Nachreiner D, Patel J, et al. Impact of standardized palliative care order set on end-of-life care in a community teaching hospital. J Palliat Med 2011;14(3):281–286; doi: 10.1089/jpm.2010.0398 21361835

[30] Walling AM, Brown-Saltzman K, Barry T, et al. Assessment of implementation of an order protocol for end-of-life symptom management. J Palliat Med 2008;11(6):857–865; doi: 10.1089/jpm.2007.0268 18715178 PMC2988455

[31] Downing DGM. Palliative Performance Scale PPSv2. n.d.;2.

[32] Anderson F, Downing GM, Hill J, et al. Palliative performance scale (PPS): a new tool. J Palliat Care 1996;12(1):5–11. 8857241

[33] Ho F, Lau F, Downing MG, et al. A reliability and validity study of the Palliative Performance Scale. BMC Palliat Care 2008;7(1):10; doi: 10.1186/1472-684X-7-10 18680590 PMC2527603

[34] IBM Corp. IBM SPSS Statistics for Windows, Version 22.0. Armonk, NY: IBM Corp. 2013.

[35] Connors AF Jr, Dawson NV, Desbiens NA, et al. A controlled trial to improve care for seriously ill hospitalized patients: The study to understand prognoses and preferences for outcomes and risks of treatments (SUPPORT). JAMA 1995;274(20):1591–1598; doi: 10.1001/jama.1995.03530200027032 7474243

[36] UpToDate. UpToDate: Discussing Goals of Care. n.d. Available from: https://www.uptodate.com/contents/discussing-goals-of-care#H106822019 [Last accessed: April 10, 2022].

[37] Bender MA, Hurd C, Solvang N, et al. A new generation of comfort care order sets: Aligning protocols with current principles. J Palliat Med 2017;20(9):922–929; doi: 10.1089/jpm.2016.0549 28537773

[38] Sutherland M, Stilos K (Kalliopi). Evaluating the pharmacological management of terminal delirium in imminently dying patients with and without the comfort measure order set. J Hosp Palliat Nurs 2019;21(5):430–437; doi: 10.1097/NJH.0000000000000585 31356358

[39] Ellerbeck EF, Bhimaraj A, Hall S. Impact of organizational infrastructure on beta-blocker and aspirin therapy for acute myocardial infarction. Am Heart J 2006;152(3):579–584; doi: 10.1016/j.ahj.2006.02.011 16923434

[40] Gardetto NJ, Greaney K, Arai L, et al. Critical pathway for the management of acute heart failure at the Veterans Affairs San Diego Healthcare System: transforming performance measures into cardiac care. Crit Pathw Cardiol 2008;7(3):153–172; doi: 10.1097/HPC.0b013e31818207e4 18791404

