# Supplementary material for: Comparing Adherence with Best Practices in End-of-Life Care After Implementing the End-of-Life Order Set: A Quality Improvement Project in an Ottawa Academic Hospital
Source: Palliat Med Rep. 2023 Apr 14;4(1):100–7. doi: 10.1089/pmr.2022.0070 (PMC10122227; doi:10.1089/pmr.2022.0070)
Supplement: Supplemental data [file Suppl_AppendixSA1.zip › Order set End of life_page1.pdf]

Montfort

HÔPITAL MONTFORT
713, chemin Montréal Road Ottawa
(Ontario) K1K 0T2
(613) 746-4621

FIN DE VIE / *END OF LIFE*
ORDONNANCES / *ORDERS*

CRITÈRES REQUIS / *REQUIRED CRITERIA*

1. **Ordonnance de 'non-réanimation' / *DNR order* (niveau C)**
2. **Patient est mourant d'une maladie terminale, pronostic jours/ heures / *Patient is dying of a life-limiting illness, prognosis hours / days (PPS plus petit ou égal 30%)*▲**
3. **Les buts de soins ont été discutés avec patient / famille / mandataire / *Goals of care have been discussed with patient / family / substitute decision maker***

DIÈTE / *DIET*

- ☐ SIPS selon tolérance / *as tolerated*
- ☐ Autre / *Other*___________________________

CONSULTATIONS

- ☐ Équipe de soins palliatifs / *Palliative Care Team*
- ☐ Service des soins spirituels et réligieux / *Spiritual and Religious Care Services*

INTERVENTIONS

- ☐ Cesser signes vitaux routines / *Discontinue routine vital signs (RR et / and T prn)*
- ☐ Insérer sonde urinaire prn / *Insert foley catheter prn* ▲
- ☐ Oxygène pour confort prn (via canule nasale) / *Oxygen for comfort prn (via nasal prongs)* ▲
- ☐ Papillons sous-cutanés prn / *Subcutaneous butterfly prn*
- ☐ Annuler laboratoires et autres examens diagnostiques / *Cancel blood work and other diagnostic exams if ordered*
- ☐ Annuler prise de glycémie capillaire / *Cancel glucose monitoring*

SOINS DE LA BOUCHE / *MOUTH CARE*

- ☐ Soins de la bouche q4h et prn / *mouth care with toothette q4h et prn*
- ☐ *Moi-Stir® mouth spray*/ou équivalent prn

HYDRATATION PARENTÉRALE / *PARENTERAL HYDRATION*▲

- ☐ Cesser soluté en cours / *Discontinue I V fluids*
- ☐ Hypodermoclyse / *Hypodermoclysis (NaCl)* __________mL / h (max 700 - 1000 mL / 24h)
- ☐ IV ______________________(solution) ________ mL / h
- ☐ Enlever robinet salin / *Remove salin lock*

FAMILLE / *FAMILY*

- ☐ Personne à contacter / Person to contact______________
  # ______________________________
- ☐ Demandes spéciales pour soins en fin de vie ou après la mort / *special requests regarding end of life care or after death care* (ex : dernier sacrement; rituels religieux spécifiques / *last rites, specific religious rites*)______________________________

MÉDICAMENTS / *MEDICATIONS* ▲

- ☐ Initier standard of care EOL
- ☐ Cesser tous médicaments préalables / *Discontinue all previous medication orders*

OU / *OR*

- ☐ Voir ordonnances médicales pour précisions sur médicaments à cesser/ *See medical orders for specification on medications to stop*

DOULEUR et / ou DYSPNÉE *PAIN and / or DYSPNEA* ▲

- ☐ Morphine _____mg SC q____h régulièrement / *regularly*
- ☐ Morphine _____mg SC q1h prn

OU / *OR*

- ☐ HYDROmorphone _____mg SC q___h régulièrement / *regularly*
- ☐ HYDROmorphone _____mg SC q1h prn

OU / *OR*

- ☐ Autres / *Other*______________________________

AGITATION / DELIRIUM: ▲ NAUSÉES / VOMISSEMENTS *NAUSEA / VOMITING*▲

- ☐ Halopéridol _____ mg SC q _____h regulièrement / *regularly*
- ☐ Halopéridol _____ mg SC q_____h prn
- ☐ Autres / *other*______________________

2ième LIGNE POUR DYSPNÉE/DÉLIRIUM / *2nd LINE FOR DYSPNEA / DELIRIUM* ▲

- ☐ Méthotriméprazine (Nozinan®) _______mg SC q______h prn (pas via un dispositif SC / *not via SC set)*
- ☐ Midazolam 1mg SC q1h prn

CONSTIPATION

- ☐ Bisacodyl 10mg IR q3jours/days prn si pas de selle / if no bowel movement

SÉCRÉTIONS / *SECRETIONS*▲

- ☐ Glycopyrrolate 0,4mg SC q2h prn

OU / OR

- ☐ Scopolamine 0,4mg SC q4h prn (↑ sédation / *sedation*)

OU / OR

- ☐ Atropine 1% Sol Oph 2 gouttes / *drops* sublingual q4h prn

FIÈVRE / *FEVER*

- ☐ Acétaminophen 650mg IR / PR q4h prn

CONVULSIONS / *SEIZURES* ▲

- ☐ Lorazepam 2 mg SC / IM / IV **STAT** – Répéter q15minutes prn et appeler médecin / *May repeat q15minutes prn and call physician* **MAX 3 DOSES**

DATE : ________________ HEURE / TIME : _____________
SIGNATURE MÉDECIN / *PHYSICIAN SIGNATURE* :
____

84500407

BLANCHE / *WHITE* **PHARMACIE/*PHARMACY*** JAUNE / *YELLOW* **DOSSIER/*CHART***

4100452 (09-16)

▲ = *Voir perles cliniques au verso / See reverse for clinical pearls*
