# Supplementary material for: Comparing Adherence with Best Practices in End-of-Life Care After Implementing the End-of-Life Order Set: A Quality Improvement Project in an Ottawa Academic Hospital
Source: Palliat Med Rep. 2023 Apr 14;4(1):100–7. doi: 10.1089/pmr.2022.0070 (PMC10122227; doi:10.1089/pmr.2022.0070)
Supplement: Supplemental data [file Suppl_AppendixSA1.zip › Order set End of life_page2.pdf]

PERLES CLINIQUES / CLINICAL PEARLS

CRITÈRES REQUIS / *REQUIRED CRITERIA*

- **Tous ces critères sont nécessaires pour utiliser ces ordonnances / *All criteria are required to use these orders***
- **PPS ≤ 30% : « Palliative Performance Scale »**
  PPS ≤ 30% = le patient est alité, nécessite des soins complets, apport per os réduite ou nul / *PPS ≤ 30% = the patient is bedridden, total care required, reduced oral intake or nothing PO*

INTERVENTIONS

- ☐ Surveiller le rythme respiratoire et la température et intervenir au besoin (ex : opiacé prn si RR rapide) / *Monitor respiratory rate and temperature and intervene as required (ex : opioids for rapid RR)*
- ☐ Patient à risque de rétention urinaire / *assess for urinary retention*
- ☐ **Oxygène pour confort prn (via canule nasale) / *Oxygen for comfort prn (via nasal prongs)***

  -Il n'existe pas une forte corrélation entre l'administration de l'oxygène en fin de vie et le confort. En fait, il est normal que la saturation d'oxygène diminue en approchant la mort. L'exception peut être le patient déjà dépendant sur l'oxygène AVANT la fin de vie – dans ce cas, viser un bon contrôle de la dyspnée, puis changer à une canule nasale (si sur masque d'oxygène).

  -There is no strong correlation between oxygen administration and comfort at end of life. In fact, it is expected that oxygen saturation will decrease as patients approach death. An exception may be with patients that are dependent on oxygen prior to reaching end of life. In these cases, consider good control of dyspnea, and then changing to nasal prongs (if on oxygen mask)

HYDRATION PARENTÉRALE/ *PARENTERAL HYDRATION*

- ☐ L'hydratation artificielle ne contribue habituellement pas au confort en fin de vie / *Artificial hydration usually does not contribute to comfort at the end of life*
- ☐ Si l'hydratation artificielle est désirée, considérer l'hydratation sous-cutanée, si l'accès veineux est difficile ou compromis/ *If artificial hydration is desired, consider subcutaneous hydration if IV access is difficult or compromised*

MÉDICAMENTS / *MEDICATIONS*

- ☐ Considérer cesser tous médicaments PO, et tous médicaments qui ne contribuent pas au confort (exemple : enoxaparin). Certains médicaments (exemple: furosémide) peuvent être continués s'ils contribuent au confort. / *Consider discontinuing all PO meds and meds not contributing to comfort (ex: enoxaparin). Certain medications (ex: furosemide) could be continued if they contribute to patient's comfort.*

DOULEUR et/ou DYSPNÉE *PAIN and/or DYSPNEA*

- ☐ Si le patient n'a ni douleur, ni dyspnée, une dose régulière n'est PAS nécessaire; considérer prn seulement / *If the patient does not have pain or dyspnea, a regular opioid dose is not necessary ; consider prn only.*
- ☐ L'intervalle habituel est de q4h; considérer q6h en insuffisance rénale / *Usual interval is q4h; consider q6h in renal failure*
- ☐ Dose de début chez patient non-tolérant aux opiacés / *Starting dose in opioid naive patient*
  - **Morphine 2 – 5 mg SC**
  - **Hydromorphone 0,25 - 1 mg SC**

Porter une attention particulière aux patients déjà sur opiacés (donc tolérants) / *Special consideration needed for patients on pre-existing opioids (therefore tolerant)*

AGITATION / DELIRIUM :

- ☐ L'utilisation d'anti-psychotiques à la plus petite dose efficace est indiquée pour un patient avec un délirium sévère agité ou avec des symptômes psychotiques / *the use of antipsychotics at the smallest effective dose is indicated for a patient with an agitated delirium or one with psychotic features*
- ☐ Dose : **Halopéridol 0,5 – 1 mg po ou sc q1h PRN ou BID reg**
- ▪ Si le délirium n'est pas bien contrôlé , considerer un agent de 2ième ligne / *If the delirium is not well controlled after 24hrs, consider a 2nd line agent*

2ième LIGNE POUR DYSPNÉE / DELIRIUM *2nd LINE FOR DYSPNEA / DELIRIUM*

- ☐ **Méthotriméprazine (Nozinan ®)**
  - 2ième ligne pour un délirium agitée malgré halopéridol (dose de début 5-10 mg SC) / *2nd line for agitated delirium, not responding to haloperidol (starting dose 5-10 mg SC)*
  -2ième ligne pour dyspnée malgré opiacés (dose de début 2,5 – 5 mg SC) / *2nd line for dyspnea despite opioids (starting dose 2,5 – 5 mg SC)*
- ☐ **Midazolam**
  -Utilisé pour gestion de crise, car action rapide mais de courte durée / *Used to control a "crisis" as quick onset but short acting*
- ▪ **Lorazepam**
  -Si une benzodiazépine est prescrite pour contrôle d'un délirium, ceci devrait être en combinaison avec un anti-psychotique (benzodiazépine seul peut augmenter un délirium) / *If a benzodiazepine is prescribed for control of a delirium, this should be in combination with an anti-psychotic (benzodiazepines alone may increase delirium)*

NAUSÉES/ VOMISSEMENTS *NAUSEA / VOMITING*

- ☐ Halopéridol est un anti-émétique puissant et utile en fin de vie / *Haloperidol is a potent anti-emetic and useful at end of life*

SÉCRÉTIONS / *SECRETIONS*:

- ☐ La scopolamine cause extrêmement de somnolence; y recourir chez les patients qui ne sont plus conscients / *Scopolamine is extremely sedating; reserve only in patients who are no longer wakeful*
- ☐ Le repositionnement du patient peut être une intervention très efficace/ *Repositioning the patient may be a more effective intervention*

CONVULSIONS / *SEIZURES*:

- ▪ Si convulsions récurrentes, considérer l'ajout d'un agent anti-convulsivant tel que le phenobarbital sous-cutanée (profond) / *If recurrent seizures, consider the addition of an anti-seizure agent such as phenobarbital given by deep subcutaneous injection*

BLANCHE / *WHITE* **PHARMACIE/*PHARMACY*** JAUNE / *YELLOW* **DOSSIER/*CHART***
